# Extrusion Characteristics of Thin Walled Tubes for Catheters Using Thermoplastic Elastomer

**DOI:** 10.3390/polym12081628

**Published:** 2020-07-22

**Authors:** Soonmo Cho, Euntaek Lee, Seunggi Jo, Gyu Man Kim, Woojin Kim

**Affiliations:** 1 Safety System R&D Group, Korea Institute of Industrial Technology, 320 Techno-sunhwan-ro, Yuga-myeon, Dalseong-gun, Daegu 711-880, Korea; holmes92@kitech.re.kr (S.C.); wessan@kitech.re.kr (S.J.); 2 School of Mechanical Engineering, Kyungpook National University, #1370 Sangyuk-dong, Buk-gu, Daegu 702-701, Korea; 3 Mechanical System Engineering, Kumoh National Institute of Technology, 61, Daehak-ro, Gumi-si, Gyeongsangbuk-do 39177, Korea; euntaek@kumoh.ac.kr

**Keywords:** polymer extrusion, thermoplastic elastomer, catheter, thin walled tube

## Abstract

As the market for minimally invasive surgery has grown, the demand for high-precision and high-performance catheters has increased. Catheters for the diagnosis and treatment of cardiovascular or cerebrovascular disease mainly use a braided wire tube with a polymer inner liner and outer jacket to improve the pushability and trackability. The outer jacket should have an accurate inner and outer diameter and while maintaining a wall thickness of 150 µm or less. In this study, we designed and manufactured a tip and die capable of extruding an outer jacket with a wall thickness of 150 µm or less using a medical thermoplastic elastomer for manufacturing 8Fr (2.64 mm diameter) thin-walled tubes. The ovality and inner/outer diameters of the tube were studied according to changes in the screw speed (mass flow rate), puller speed, air pressure applied to the lumen, and distance between the quench and head, which are the main variables of microextrusion processes. The screw speed (mass flow rate), puller speed, and air pressure affected the inner/outer diameter of the tube, with screw speed and puller speed having the largest influence on diameter. The air pressure and distance between quench and head had the greatest influence on ovality. The results show the effect of different processing parameters on the characteristics of the extruded tube, which will help to establish a stable extrusion process for the manufacture of outer jackets for braided catheter shafts.

## 1. Introduction

Catheters are minimally invasive interventional medical devices actively used in numerous procedures, including stent delivery, drug and contrast injection, imaging guide diagnosis, and ablation [1,2,3]. These thin tubes are generally 300–2000 mm long, with a single-lumen (cavity in the tube) or multi-lumen cross-section and a braided or coiled shaft to meet the demands of the application. During use, the distal portion of the catheter (the far end) is steered unidirectionally or bidirectionally [4,5,6,7,8]. Today, catheters are commonly fabricated from thermoplastic elastomers [9,10] through an extrusion process [11,12,13,14]. In the medical device industry, the outer diameter of the catheter shaft is typically less than 3 mm, while the interventional catheters used in cardiovascular and cerebrovascular fields have outer diameters of 1 mm or less [15,16,17]. Therefore, extrusion systems are required that can produce tubes with smaller diameters than those typically manufactured in other industries. Extrusion systems have many components, including a dryer and dehumidifier, hopper, screw and barrel, tip and die, quenching system, vacuum water tank, measurement device, puller, cutter, and conveyor system. The polymer is dried and dehumidified before being injected through the hopper of the extruder into the screw and barrel, where it melts from the heat of friction among the screw, polymer, and inner wall of the barrel. The initial shape of the tube is formed as the polymer passes through the tip and die. The pressure of the air injected into the lumen. As extrusion continues, the tube is slightly hardened by quenching, and the lumen of the tube is stably retained as it passes through the vacuum tank filled with water. The tube is pulled during extrusion and cut to the desired length in the conveyor system. The measuring device is used to measure the wall thickness and tube diameter during extrusion.

As the market for minimally invasive surgery has grown, the demand for high-precision and high-performance catheters has increased. Catheter manufacturing techniques have advanced over time, although more precise catheter manufacturing techniques are still required. In addition, microextrusion techniques are being actively studied to produce steerable or smart catheters [18,19,20,21]. Column strength, flexibility, and buckling are important mechanical properties for sheath catheters used for stent delivery and electrophysiology catheters for intervention of arrhythmia and atrial fibrillation. These catheters typically have braided shafts [22,23,24]. An elastomeric tube with thin and uniform walls is used as an outer jacket of braided shafts. The quality of the thin and uniform wall thickness for the out-jacket tube influences the stable mechanical performance of the braided shaft. In general, the wall thickness of the outer jacket is about 0.1–0.2 mm, so even if the tube is single-lumen, the rheology of the polymer melt and the size and shape of the tip and die are very important [25]. Furthermore, the relationships between the polymer melt flow rate, the pressure of the air injected into the lumen, the puller speed, and the quenching conditions must be understood clearly. However, not much research has been conducted for this purpose so far.

In this work, we studied the extrusion process for elastomer tubes with a wall thickness of 150 μm or less for the outer jacket of braided catheter shafts used in cardiovascular and cerebrovascular intervention. The tip and die were designed considering the rheological properties of the polymer, the head pressure, and die swelling. In addition, the shape and ovality of the tube was analyzed changing the distance between the end of the tip and the quenching region, the screw speed, the puller speed, and the pressure of air injected into the lumen. Wall thickness is a major factor affecting the quality of concentricity of a catheter body, and the precision of the inner and outer diameter is important for surface roughness, air bubble generation, and strain line generation of the braided tube. Finally, the main parameters required to stably manufacture thin-walled tubes with the desired dimensions was derived.

## 2. Experimental Methods

### 2.1. Materials and Numerical Analysis

**Figure 1:**
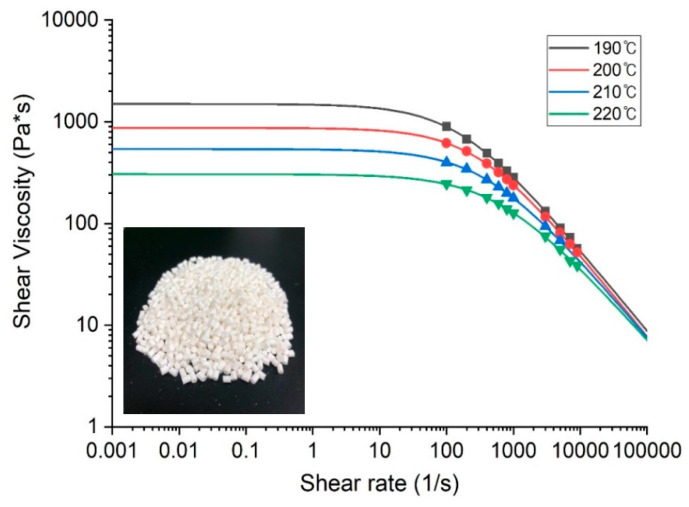
Variation in viscosity according to shear rate (Pebax 4033 MED SA01 + 20% BaSO_4_).

We used polyether block amide Pebax 4033 SA MED pellets (Arkema, France), which is a representative biocompatible elastomer that is actively used in catheter manufacture. The mechanical properties of Pebax4055 are shown in Table 1. The polymer was compounded with 20 wt% BaSO_4_ to provide radiopacity. This elastomer is used in the distal part of endovascular and steerable catheters, in conjunction with a relatively rigid proximal shaft. The viscosity of viscoelastic fluids, including medical polymer melts, generally differs depending on the temperature and shear rate. As shown in Figure 1, to analyze this phenomenon, the viscosity of the polymer according to the shear rate in the temperature range of 190–220 °C was analyzed using a capillary rheometer (Rheograph 75, Göttfert, Germany). To obtain stable rheological curve fitting, we used the Bird–Carreau model (Equation (1)), which is a generalized Newtonian model, and the Arrhenius model (Equation (2)), considering the isothermal conditions [5].

(1) $$\eta \left(\dot{\gamma }\right)=\eta_{\infty }+\left(\eta_{0}-\eta_{\infty }\right){\left(1+\lambda^{2}{\dot{\gamma }}^{2}\right)}^{\frac{n-1}{2}}$$

where *η*_∞_ is the viscosity at infinite shear rate, *η*_0_ is the viscosity at zero shear rate, *λ* is the relaxation time, $\dot{\gamma }$ is the local shear rate, and *n* is the power law index.

(2) $$H\left(T\right)=\exp\left[\alpha \left(\frac{1}{T-T_{0}}-\frac{1}{T_{\alpha }-T_{0}}\right)\right]$$

where *α* is the ratio of the activation energy to the perfect gas constant, and *T_α_* is the reference temperature for which *H*(*T*) = 1. The temperature shift *T*_0_ is set to 0 by default, and corresponds to the lowest thermodynamically acceptable temperature for extrusion. Therefore, *T* and *T*_α_ are absolute temperatures. They can also be defined relative to a non-absolute temperature scale, in which case *T*_0_ corresponds to the lowest temperature of the current temperature scale. Equation (2) represents the correlation between temperature and shear rate, and the input values for each model are shown in Table 2.

Figure 1 also shows the change in viscosity as function of the shear rate and temperature. As expected, the viscosity decreases with an increase in the shear rate (shear thinning behavior) or an increase in temperature. Especially, when the shear rate was over 100 [1/s], the viscosity decreased rapidly in all temperature ranges, and the viscosity also decreased at the same shear rate as the temperature increased. The tip and die structures based on the rheological properties of the polymer was optimized, considering various draw ratios, such as the diameter draw ratio (DDR; Equation (3)), wall draw ratio (WDR; Equation (4)), and sizing ratio (SR; Equation (5)) [15].

(3) $$DDR=\frac{D_{t}+D_{d}}{D_{o}+D_{i}}$$

(4) $$WDR=\frac{D_{d}-D_{t}}{D_{o}-D_{i}}$$

(5) $$SR=\frac{WDR}{DDR}$$

where *D*_t_ and *D*_d_ are the outer diameter of the tip and inner diameter of the die, respectively; *D*_o_ and *D*_i_ are the outer and inner diameter of the extruded tube, respectively. 

In the process of designing the tip and die, the SR value and the allowable pressure of the extruder used in this study were considered. When the SR value is smaller than 1, it is difficult to extrude the tube to a uniform size, and when it is larger than 1.3, the roughness of the tube surface may increase or tearing may occur. [15] Therefore, we designed the outer diameter of the tip and the inner diameter of the die to maintain an SR of 1.15. Furthermore, the rheological properties were considered to control the pressure of the polymer melt to 35 MPa or below, which is the stable range of head pressure of the extruder used in this study. 

The flow of the polymer melt was analyzed using the rheological data of the polymer (Figure 1) and Ansys Polyflow^®^. The die swell phenomenon can be minimized by uniformizing the velocity of the polymer melt within the maximum head pressure of the extruder cylinder. 

**Figure 2:**
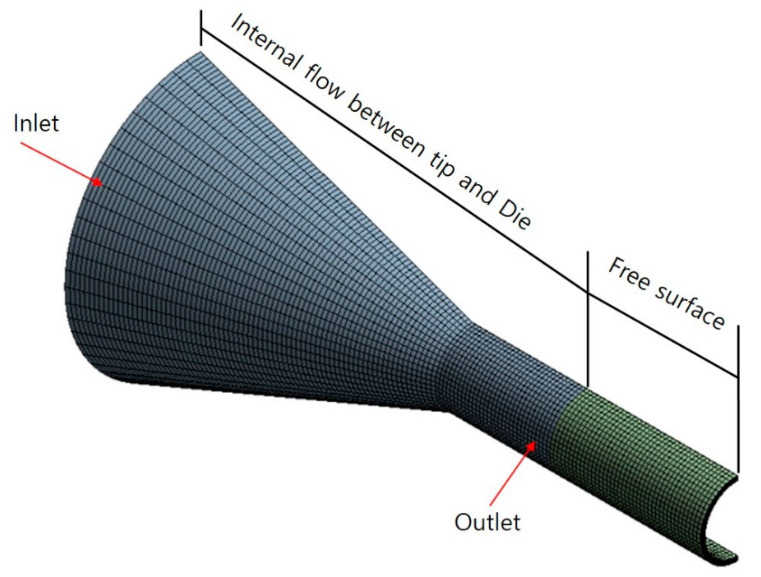
Mesh and boundary conditions of the numerical analysis.

Figure 2 shows the shape of the three-dimensional model and boundary conditions for the internal flow path of the polymer melt and mesh for numerical analysis. The model comprises 50,809 nodes and 44,400 elements. For the inlet, an initial mass flow rate of 0.108 g/s was applied, which is the same as that of the extruder, and no slip conditions were applied to either the inner surface of the die or the surface of the tip. In addition, we modeled the free surface section between the end of the tip and die to the quenching section, setting a length of 10 mm. The model results were observed according to the pulling force on the extruded tube.

### 2.2. Experimental Setup

**Figure 3:**
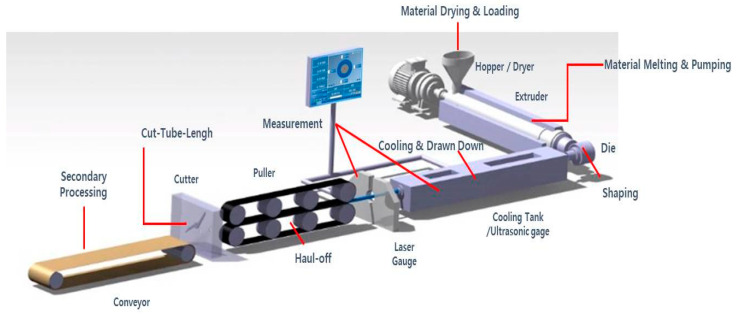
Experimental setup of microextrusion process.

The microextrusion system (see Figure 3) has a single-screw extruder (Davis-Standard, USA) with a screw diameter of 25.4 mm and length–diameter ratio of 25:1. A barrier screw with a spiral Maddock mixer was used, and detailed specifications of the screw are shown in Table 3. A vacuum tank, air injection system, puller, cutter, and conveyor were mounted as downstream equipment. The Pebax pellets were dried for 4 h at approximately 60 °C by hot air in the dryer before injection through the extruder hopper. The flow rate of the polymer melt was controlled by adjusting the rotational speed of the screw, and the shape and size of the final tube was determined by the drawing process and sizing process in the vacuum tank. 

The tip and die were designed to give a 2.3/2.6 mm (i.d./o.d.) medical tube, and manufactured through lathe machining and EDM (electric discharge machining) processing. The temperatures of the barrel, head, and die were controlled as shown in Table 4 in order to manufacture the tube. The screw speed (flow rate of polymer melt), puller speed, air pressure, and distance between the tip and quenching system were set as shown in Table 5. Moreover, the changes in the inner and outer diameter and ovality of the tube was observed according to four process parameters (see Table 5): pressure of air injected into the lumen (0.19–1.96 kPa); distance between tip and quenching (10–100 mm); screw speed (4–11 rpm); and puller speed (5.3–9.3 m/min). In addition, the size and ovality of the tube were monitored on-line using an ultrasonic diameter gauge (Ultrascan 1000, Beta laser mike, Dayton, Ohio, USA) to measure the wall thickness, and a 4-axis laser diameter and ovality gauge (Acuuscan 6012, Beta laser mike, Dayton, Ohio, USA) to measure the outer diameter and ovality. The final inner and outer diameter and ovality were also repeatedly were measured off-line at multiple positions using a coordinate measuring machine (O-INSPECT 543, Carl Zeiss, Oberkochen, Germany). The ovality was defined as follows [26]:

(6) $$\sqrt{\frac{\sum_{1}^{N}{\left(r-\mu \right)}^{2}}{N}}$$

where *r* is the radius at a given position, *N* is the total number of positions in one rotation, and *μ* is the mean radius of the tube.

Consequently, the major factors affecting the tube size and ovality was analyzed during the manufacture of a tube with a wall thickness of 150 μm or less.

## 3. Results and Discussion

### 3.1. Tip and Die Manufacture Based on Ratio Theories and Numerical Analysis

**Figure 4:**
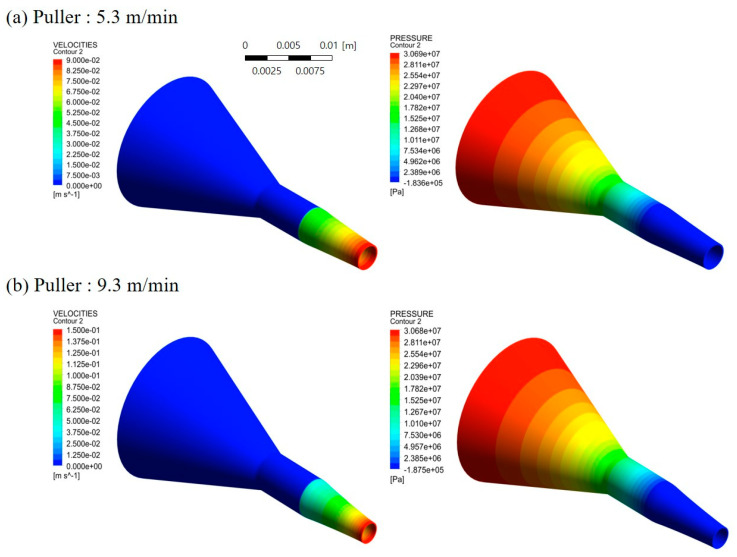
Velocity and pressure profiles of the polymer melt between the tip and die at puller speeds of (**a**) 5.3 m/min and (**b**) 9.3 m/min.

**Figure 5:**
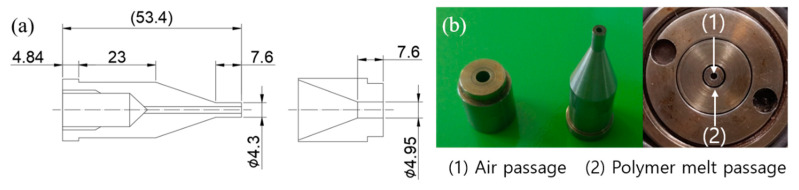
**(a)** Optimized design of tip and die and (**b**) manufactured tip and die.

Figure 4 shows the results of analyzing the flow velocity and pressure profiles of the polymer melt between the tip and die according to the puller speed, considering the allowable die pressure and SR value of the extruder used in this study. The puller speed was varied between 5.3 and 9.3 m/min, and the flow rate of polymer melts was fixed at 15.84 g/min which is the flow rate at screw speed of 11 rpm. It was confirmed that the pressure of the polymer melt was less than or equal to 35 MPa with a tip outer diameter and die inner diameter of 4.3 and 4.95 mm, respectively. As the puller speed was increased, the velocity of the polymer melt in the free surface region increased. Nevertheless, a uniform velocity profile was observed, indicating that tube instability caused by die swell would be small. Based on these results, a tip and die were manufactured as shown in Figure 5. The outer diameter of the tip (*D*_t_) and inner diameter of the die (*D*_d_) were 4.3 and 4.95 mm, and DDR and SR were 1.89 and 1.15, respectively.

### 3.2. Analysis on the Flow Rate of Polymer Melt and Stability of Tube Manufacturing

**Figure 6:**
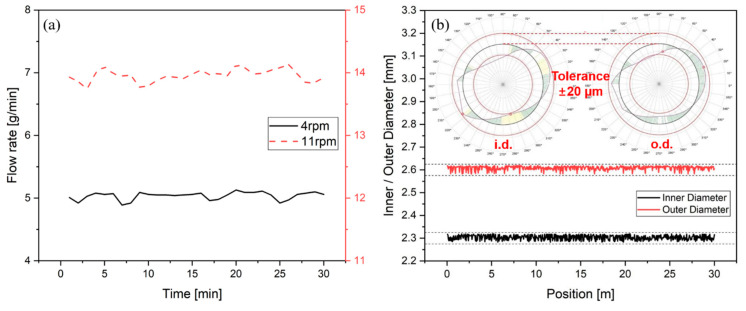
(**a**) Variation in flow rate of polymer melt over time at 4 and 11 rpm and (**b**) variation of the inner diameter and outer diameter of the extruded tube in real time under the reference manufacturing conditions.

When the extrusion with the manufactured tip and die was performed, the flow rate of the polymer melt was measured and the stability of the tube extrusion was investigated. Figure 6a shows the measured variation of the flow rate of polymer melt over time at 4 and 11 rpm which are the minimum and maximum screw speeds of this study. The flow rate of the polymer melt was uniform and the tolerance was about ±0.2 g/min or less based on 30 min experimental measurement for both screw speeds. Furthermore, the manufactured tip and die were manufactured to maintain uniform flow rates. Figure 6b shows the results of measurement the inner diameter and outer diameter of the tube extruded in real time under the reference manufacturing conditions of the study. In addition, the wall thickness of the extruded tube using a coordinate measuring machine was shown in Figure 6b. The tolerance between the inner diameter and the outer diameter of the tube was observed at the level of ±20 um, and the wall thickness of the tube did not exceed the tolerance of ±15 um.

### 3.3. Variation of Tube Diameter and Ovality with Air Pressure 

**Figure 7:**
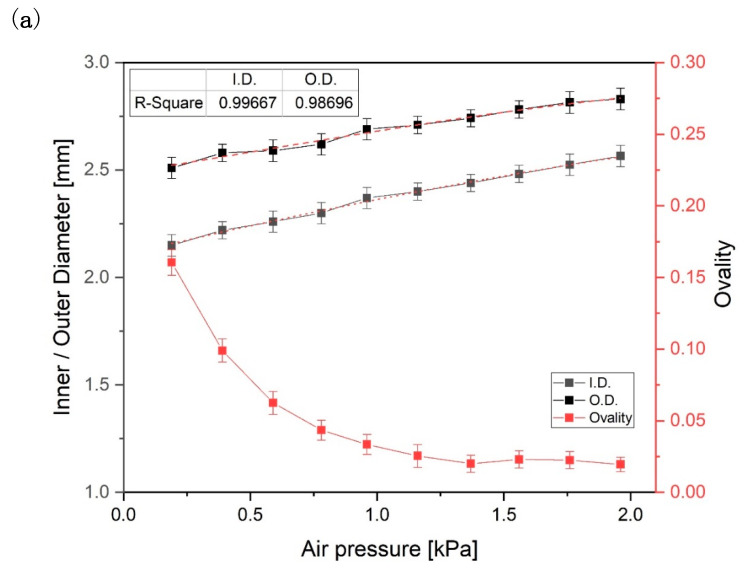

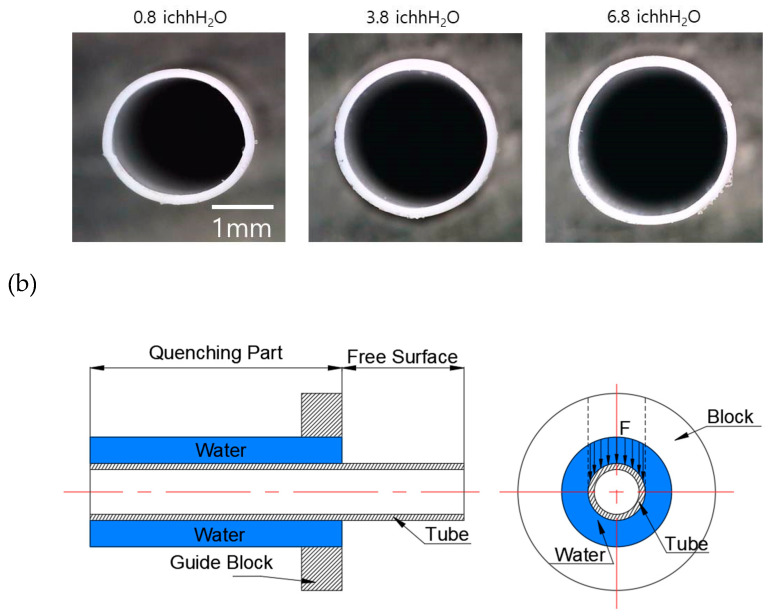
(**a**) Variation of inner/outer diameter and ovality of the tube with air pressure; (**b**) effect of cooling water layer on the extruded tube. The p-values of each section ovality are 0.005–0.01 compared to the ovality value of 0.19 kPa.

The effects of different processing parameters on the tube dimensions (inner/outer diameter) and ovality were investigated using the tip and die described in Section 3.1. Figure 7a shows the effect of the pressure of the air injected through the inside of the tip, with cross-sectional images of the tubes produced under each condition. As the air pressure increases from 0.19 to 1.96 kPa in 0.2 kPa increments, the inner and outer tube diameter increased. The ovality also gradually improved until about 4 in H_2_O, where it reached a constant minimum of 0.025. These results demonstrate that the radial force inside the tube increases as the air pressure increases. The force affects the polymer chain entanglement before the tube polymerizes. As a result, the air pressure flowing through the tip has a significant influence on the inner and outer diameters of the tube. However, the wall thickness was slightly reduced from 0.18 to 0.14 mm as the air pressure increased, because the flow rate of the polymer melt does not change.

Meanwhile, when low air pressure is injected through the tip, a low radial force is applied to the tube that does not overcome the load of the cooling water layer surrounding the tube at the guide block, which induces poor ovality. Thus, the ovality of the tube improves as the air pressure increases as it is able to withstand the load of the cooling water layer.

### 3.4. Variation of Tube Diameter and Ovality with Distance between Tip and Quenching Part 

**Figure 8:**
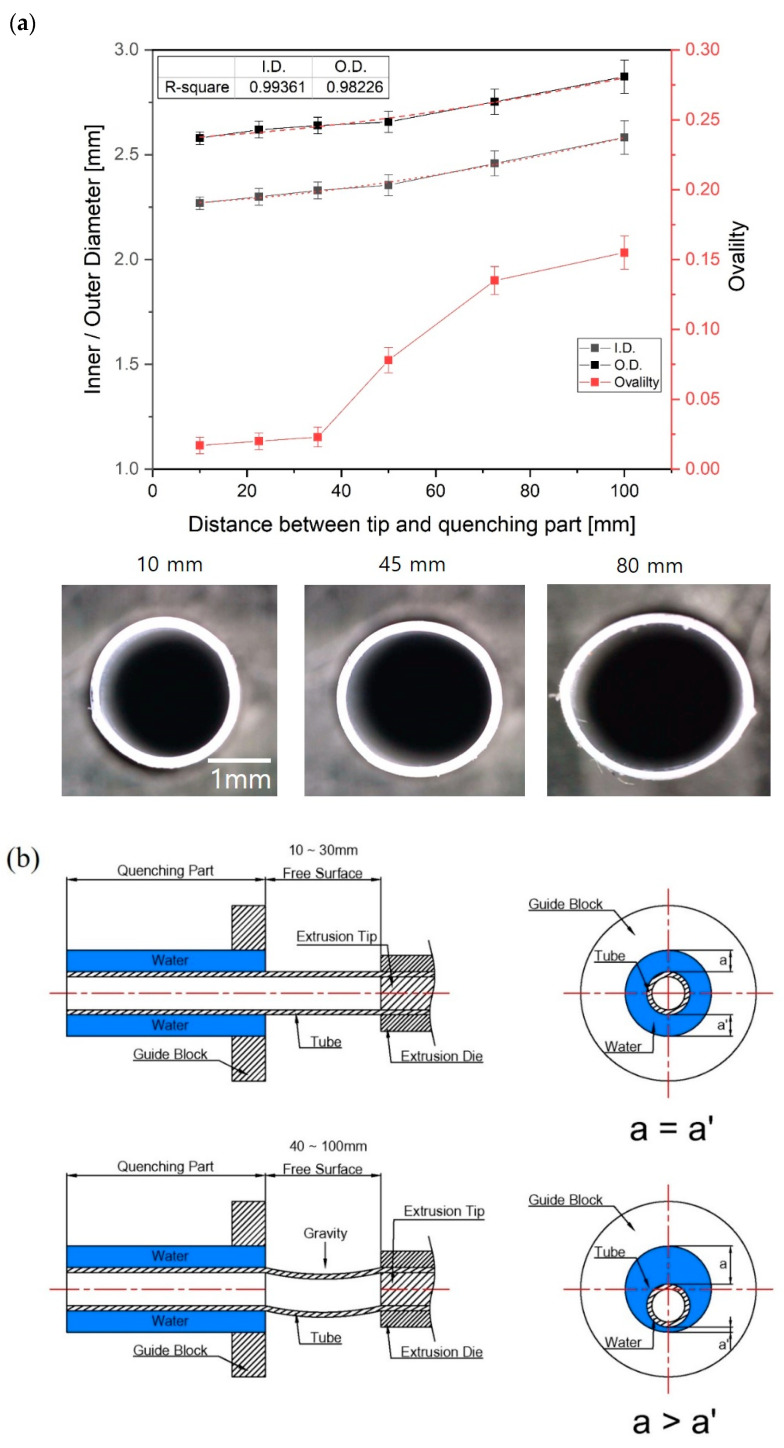
(**a**) Variation of inner/outer diameter and ovality of the tube with distance between tip and quenching part and (**b**) Comparison of tube alignment at the entrance of quenching part according to distance between tip and quenching part. The p-values of each section ovality are 0.015–0.002 compared to the 10mm ovality value.

Figure 8 shows how the inner/outer diameter and ovality of the tube changed according to the distance between the end of the tip and die and the quenching region. The quenching system cools and solidifies the tube after it passes through the free surface region. As the distance was reduced from a reference distance of 30 mm, the inner and outer diameter of the tube gradually reduced and the ovality was slightly improved, but the changes were not significant. On the other hand, when the distance was increased above 30 mm, the inner and outer diameters slightly increased and the deviation of the inner and outer diameters greatly increased. Notably, the tube was not extruded stably due to the rapid rise in ovality. When the distance between the tip and quenching part was more than 60 mm, the ovality exceeded 0.1, and the cross-section of the tube was elliptical, as shown in Figure 8.

In order to examine the cause of the deviation of the inner and outer diameters and the poor ovality, we videoed the extrusion process (see Video S1 of the Supplementary Materials). When the distance between tip and quenching region was short, the extruded tube moved stably through the free surface region. However, the vibration of the tube was elevated as the distance increased, and the cooling water layer surrounding the tube was also unstable when the tube entered the quenching region; therefore, this phenomenon affected the sizing process of the tube. In addition, the tube is affected by gravity and sags in the direction of gravity as the distance between tip and quenching part increases. Due to this phenomenon, when the tube enters the quenching part, the fluctuation of water layer occurs because the thickness of the surrounding water layer is uneven. Consequently, the distance between the tip and quenching part is very important for minimizing the ovality. 

### 3.5. Variation of Tube Diameter and Ovality with Screw and Puller Speed

**Figure 9:**
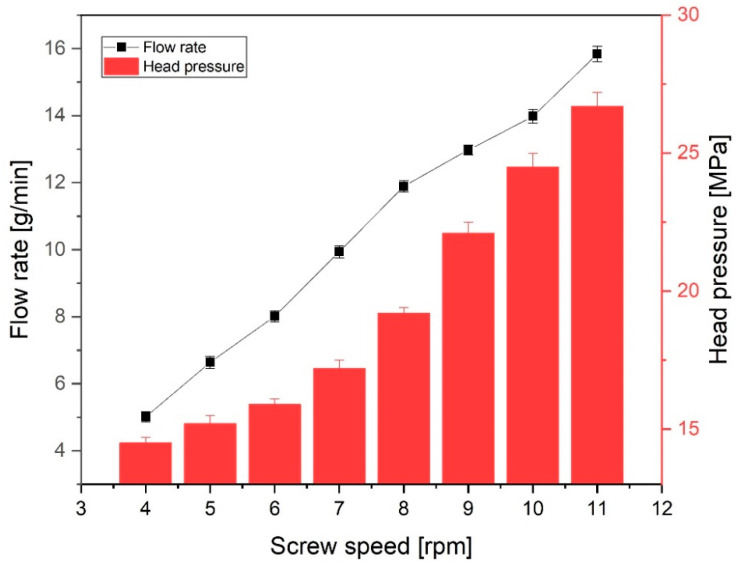
Mass flow rate of polymer melt and head pressure with screw rotation speed.

**Figure 10:**
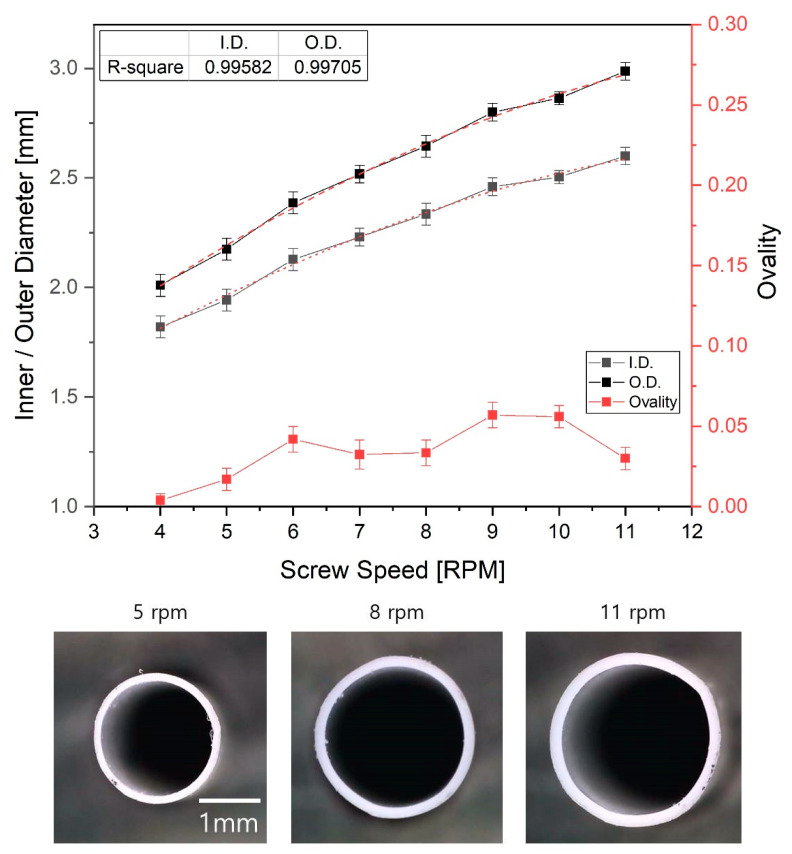
Variation of inner/outer diameter and ovality of the tube with screw rotation speed. The p-values of each section ovality are 0.003~0.01 compared to the ovality value of 4 rpm.

The tube size can be affected by the mass flow rate of the polymer melt. The mass flow rate of the polymer melt was investigated as the screw speed increased by 1 rpm because the mass flow rate is closely related to both the puller speed and screw rotation speed. The results are as shown in Figure 9. As the screw speed increases from 4 to 11 rpm in 1 rpm increments, the mass flow rate increased linearly from 5.12 to 15.84 g/min, which was expected to affect the inner/outer diameter and ovality of the extruded tube. In addition, the pressure at the head mounted with the tip and die was measured as the screw speed increased. The pressure was measured from 14.1 to 26.7 MPa while increasing from 4 rpm to 11 rpm. The pressure predicted in numerical analysis was higher than experimentally measured pressure because the point of experimentally measured pressure was at the beginning of the dip and die. The point pressure predicted in numerical analysis is at the middle of tip and die so that the pressure at the location is higher than that of the beginning of the tip and die. Therefore, the experimental results were similar to the numerical analysis results. Figure 10 shows the shape of the extruded tube at different screw speeds. The puller speed was fixed at 7.3 m/min. When the screw speed was 8 rpm, the inner/outer diameters of the tube were 2.3/2.6 mm, and the wall thickness was 0.155 mm. As the screw speed decreased to 5 rpm, these values decreased linearly to 1.8/2.0 mm and 0.095 mm, respectively. Similarly, when the screw speed increased to 11 rpm, these values increased linearly to 2.6/3.0 mm and 0.19 mm, respectively. Although the flow rate decreases as the screw speed decreases, the tube was drawn at the same speed by the puller, so the polymer melt was stretched further and solidified with a relatively thinner wall thickness. On the other hand, when the screw speed was increased, the flow rate of the polymer melt increased, so a relatively thick polymer layer was formed, resulting in increased wall thickness. Nevertheless, the change in ovality due to the screw speed was not large (see Figure 10); the ovality was maintained at 0.01–0.06 for screw speeds of 4–11 rpm.

**Figure 11:**
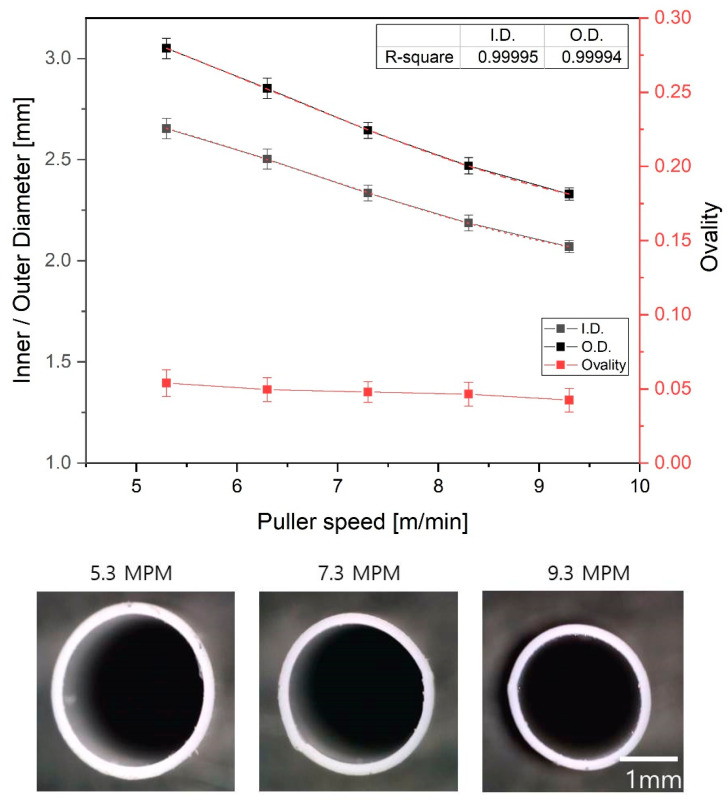
Variation of inner/outer diameter and ovality of the tube with puller speed. The p-values of each section ovality are 0.001 to 0.043 compared to the ovality value of 5.3 m/min.

Figure 11 shows the inner/outer diameter and wall thickness of the extruded tube according to the puller speed. The screw speed was fixed at 8 rpm, while the puller speed was altered between 5.3 and 9.3 m/min in 1m/min increments. At the minimum puller speed of 5.3 m/min, the inner and outer diameters of the tube were 2.65 and 3.04 mm, respectively, while the wall thickness was 0.197 mm. On the other hand, at the maximum puller speed of 9.3 m/min, these values increased to 2.50/2.31 mm and 0.13 mm, respectively. In other words, as the puller speed increased, the tube size and wall thickness decreased simultaneously. The change in ovality with puller speed was similar to that with screw speed; the overall change in ovality was small and the ovality was maintained below 0.05 (see Figure 11). As mentioned in Section 3.4, when the puller speed becomes larger than the surface velocity of the tube discharged to the free surface through the tip and die, the tube in the elastic region is stretched by the pulling force before undergoing solidification, thereby decreasing the overall tube size.

**Figure 12:**
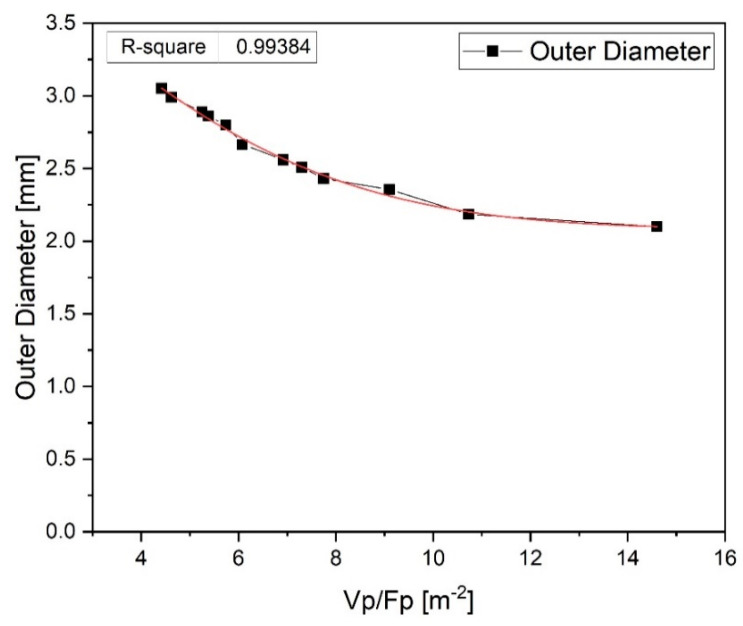
Variation in the outer diameter of the tube according to the change of *V*_p_/*F*_p_.

According to previous study [4], the diameter of the tube is affected by the ratio of the puller speed (*V*_p_) to the flow rate (*F*_p_) controlled by the screw speed. Therefore, as shown in Figure 12, the variation of the outer tube diameter according to the change of *V*_p_/*F*_p_ [m^−2^] was analyzed. The outer diameter of the tube gradually decreased as *V*_p_/*F*_p_ increased. These results were similar to the previous study [4]. As a result, a tube with an outer diameter of about 2.0 to 3.0 mm was be stably manufactured under proposed *V*_p_/*F*_p_ conditions according to the specifications of the tip and die and the temperature of the extruder in this study.

## 4. Conclusions

In this study, we developed a tip and die for the production of medical tubes with a wall thickness of 150 µm or less. The size and structure of the tip and die were determined in consideration of the allowable pressure inside the extruder and SR value, and numerical analysis was conducted to verify that a uniform velocity profile was formed near the outlet of the tip and die. 

In order to manufacture 2.3/2.6 mm (i.d./o.d.) tubes fabricated from Pebax 4033 SA01 MED + 20 wt% BaSO_4_ using the manufactured tip and die, we established reference conditions by changing the air pressure, distance between tip and quenching region, screw speed (melt flow rate), and puller speed. We then analyzed the inner and outer diameter, ovality, and wall thickness of the extruded tube while changing the main parameters of the extrusion process. The following conclusions are proposed:

1. The air pressure, screw speed, and puller speed had the largest effect on the tube size as in other conventional extrusions process. When the air pressure in the lumen increases, the inner and outer diameter increase significantly, and the ovality improves gradually. The wall thickness is slightly reduced as the air pressure increases because of the radial forces generated inside the tube. In addition, the inner and outer diameters of the thin-walled tubes were most affected by the screw rotation speed and puller speed.

2. The distance between tip and quenching region greatly influences the ovality of the extruded tube. As the distance between the tip and quenching region increases, the cooling water layer surrounding the tube at the beginning of the quenching part becomes unstable and the vibration of the water layer increases. In addition, the partially solidified tube is affected by gravity as the distance of the free surface region increases. As a result, the variation of the inner and outer diameter of the tube increase and the ovality performance decreases. 

Understanding the size and ovality characteristics of extruded tubes according to changes in the extrusion process parameters will help to develop manufacturing conditions for the stable extrusion of thin-walled tubes (below 150 μm), which are the main components of braided catheter shafts.

In this article, we focused on finding the main process variables that affect tube size and ovality in tube extrusion processes for catheters with a wall thicknesses of less than 150 um. In future work, we will compare the results of this study with respect to changes of the extruder temperature, screw speed, and puller speed, based on industry conditions.

## Figures and Tables

**Table 1:** Mechanical properties of Pebax4033 SA01 MED+20wt% BaSO_4._

Property	Value
Density	1000 [kg/m^3^]
Melting point	160 [℃]
Flexural modulus	81 [Mpa]
Tensile strength	40 [Mpa]
Melt flow index (MFR)	9 [g/10 min]

**Table 2:** Properties of Pebax 4033 SA01 MED + 20 wt% BaSO_4_ for numerical analysis.

**Bird-Carreau model**	
**Parameters**	**Value**
$\eta_{0}$ = Zero –shear rate viscosity	960.840 [Pa.s]
$\eta_{\infty }$ = Infinite –shear rate viscosity	8.09 × 10^−6^ [Pa.s]
λ = Natural time	0.006 [s]
n = Cross law index	0.296 [–]
**Arrhenius law model**	
**Prameters**	**Value**
α The ratio of the activation energy to the perfect gas constant	5677.69 [1/℃]
$T_{\alpha }$ Reference temperature	263.99 [℃]
$T_{0}$ Temperature shift	−273.15 [℃]

Density: 1000 [kg/m^3^]; heat capacity per unit (CP): 2800 [J/kg·°C]; thermal conductivity(K): 0.18 [W/m·°C].

**Table 3:** Specification of the extruder screw.

Specification of the Extruder Screw	
Composition	Specification
Total length	25 D
Length of Feed section	4.5 D
Length of metering section	10.0 D
Length of spiral mixer	2.0 D
Flight width	0.15 D
Channel depth	0.20 D
Helix angle	18.50 °

D = Screw diameter = 25.4 mm.

**Table 4:** Temperature of extruder.

	Barrel 1	Barrel 2	Barrel 3	Head	Tip & Die
Temperature [℃]	180	190	195	195	190

**Table 5:** Extrusion processing conditions of reference tubes [2.3/2.6 mm (i.d./o.d.)] and Ranges of process variables.

Process Parameter	Reference Condition	Range of Process Variables
Air injection velocity in lumen q [kPa]	0.78	0.19–1.96
Distance between tip and quenching part d [mm]	30	10–100
Screw speed n [r/min]	8	4–11
Puller speed ν [m/min]	7.3	5.30–9.30

## Supplementary Materials

The following are available online at https://www.mdpi.com/2073-4360/12/8/1628/s1, Video S1: The distance between tip and quenching part.
